# Isotopic source signatures of stratospheric CO inferred from in situ vertical profiles

**DOI:** 10.1038/s41612-025-00986-1

**Published:** 2025-03-18

**Authors:** Joram J. D. Hooghiem, Sergey Gromov, Rigel Kivi, Maria Elena Popa, Thomas Röckmann, Huilin Chen

**Affiliations:** 1 Centre for Isotope Research (CIO), Energy and Sustainability Research Institute Groningen (ESRIG), University of Groningen, Groningen, The Netherlands; 2 Atmospheric Chemistry Department, Max Planck Institute for Chemistry, Mainz, Germany; 3 Space and Earth Observation Centre, Finnish Meteorological Institute (FMI), Sodankylä, Finland; 4 Institute for Marine and Atmospheric Research Utrecht, Utrecht University, Utrecht, The Netherlands; 5 Joint International Research Laboratory of Atmospheric and Earth System Sciences, School of Atmospheric Sciences, Nanjing University, Nanjing, China; 6 Present Address: Meteorology and Air Quality, Wageningen University and Research, Wageningen, The Netherlands

**Keywords:** Biogeochemistry, Atmospheric chemistry

## Abstract

The stratospheric CO budget is determined by CH_4_ oxidation, OH-driven loss and atmospheric transport. These processes can be constrained using CO mole fractions and isotopic compositions, with the latter being largely unexplored. We present novel stratospheric observations of δ^13^C-CO and δ^18^O-CO vertical profiles, revealing distinct altitude-dependent trends. δ^13^C-CO decreases with altitude due to inverse ^13^C kinetic fractionation in the OH sink and ^13^C-depleted CO from CH_4_ oxidation. In contrast, δ^18^O-CO increases with altitude, driven by ^18^O-rich oxygen from O(^1^D) via O_3_ photolysis and CO_2_ photolysis. Our findings suggest that CO isotopes can act as valuable proxies for quantifying CO production from CO_2_ photolysis. Incorporating CO mole fractions and isotopic data into global models enhances evaluations of the stratospheric CH_4_ sink and OH abundance, improving our understanding of stratospheric water vapour and its radiative impacts.

## Introduction

Carbon monoxide (CO) is important for atmospheric chemistry and climate, mainly via the reaction with the hydroxyl radical (OH)^1,2^. Its anthropogenic sources include combustion of fossil fuels and biomass burning, which account for about half of its atmospheric budget, while the other half is from atmospheric oxidation of methane (CH_4_) and other hydrocarbons^3^. The reaction of CO with OH is the only significant sink of CO in the atmosphere.

Owing to the elemental mass difference, physical and chemical processes occur at different rates for different isotopes (or isotopologues). This phenomenon, known as isotopic fractionation, results in distinct isotope ratios (or signatures) for various sources and their subsequent modification in sink processes. Known signatures of typical sources and sinks were recently summarised by Vimont et al.^4^ and Dasari et al.^5^. For example, CH_4_ oxidation usually produces CO with lowest δ^13^C and δ^18^O values, and high temperature combustion processes generally produce CO with higher δ^18^O^6–8^. Despite being usually small, the variations in these signatures allow for quantification of source strengths using end-member mixing models^5,9–11^. Since these variations are typically small, they are expressed as a relative difference from a reference. For CO, the standard references are VPDB for ^13^C and VSMOW for ^18^O.

1 $$\mathrm{\delta ^{13}C{-}{CO}=\frac{{13\atop }CO}{{{12\atop }CO}_{{sample}}}\left/\frac{{13\atop }CO}{{{12\atop }CO}_{{reference}}}{-}1\right.}$$

2 $${\updelta}^{18}{\mathrm{O}}{-}{\mathrm{CO}}=\frac{{\mathrm{C}}{18\atop}{\mathrm{O}}}{{{\mathrm{C}}{16\atop}{\mathrm{O}}}_{\mathrm{sample}}}\left/\frac{{\mathrm{C}}{18\atop}{\mathrm{O}}}{{{\mathrm{C}}{16\atop}{\mathrm{O}}}_{\mathrm{reference}}}{-}1\right.$$

The variations in the isotope ratio can be used to identify and partition the sources of atmospheric CO^12^. However, such applications often suffer from insufficient knowledge on the isotope compositions of various sources. For example, the oxygen isotope source signature of CO from CH_4_ oxidation is uncertain, which complicates the application of isotope data as an emission or source constraint.

A secondary application of CO isotope data is investigating the loss of CH_4_ in the stratosphere. Contrary to the troposphere, where the main sink of CH_4_ is the reaction with OH, there are additional loss terms of CH_4_ in the stratosphere:

$${{\rm{CH}}}_{4}+{\rm{OH}}\rightarrow {{\rm{CH}}}_{3}+{{\rm{H}}}_{2}{\rm{O}}, \quad {\rm{R}}1$$

$${{\rm{CH}}}_{4}+{\rm{Cl}} \rightarrow {{\rm{CH}}}_{3}+{\rm{H}}{\rm{Cl}}, \quad {\rm{R}}2$$

$${{\rm{CH}}}_{4}+{\rm{O}}({}^{1}{\rm{D}}){\rightarrow {\rm{CH}}}_{3}+{\rm{OH}}, \quad {\rm{R}}3{\rm{a}}$$

$${{\rm{CH}}}_{4}+{\rm{O}}({}^{1}{\rm{D}})\rightarrow {{\rm{CH}}}_{3}{\rm{O}}+{\rm{H}}, \quad {\rm{R}}3{\rm{b}}$$

$${{\rm{CH}}}_{4}+{\rm{O}}({}^{1}{\rm{D}}){\rightarrow {\rm{CH}}}_{2}{\rm{O}}+{{\rm{H}}}_{2}, \quad {\rm{R}}3{\rm{c}}$$

$${\rm{CO}}+{\rm{OH}} {\rightarrow {\rm{CO}}}_{2}+{\rm{H}}, \quad {\rm{R}}4$$

$${{\rm{CH}}}_{3}+{{\rm{O}}}_{2} {\rightarrow {\rm{CH}}}_{3}{{\rm{O}}}_{2}, \quad {\rm{R}}5$$

$${{\rm{CO}}}_{2}+{\rm{\gamma }} \rightarrow {\rm{CO}}+{\rm{O}} \quad {\rm{R}}6$$

The carbon kinetic isotope effects of these reactions are well known^13^, and using these reactions, observations of CO depleted in ^13^C in the high southern latitude lower stratosphere could be explained^14,15^. Note that reactions following R1–3 do not affect ^13^C/^12^C of CO as the intermediate products are rapidly and completely oxidised to CO. Reaction R5 is crucial in defining the δ^18^O signature of CO produced from CH_4_ oxidation, as pointed out by Weston (2001)^16^.

Previous attempts to measure the CO isotopic composition at altitudes above 12 km in the stratosphere have been unsuccessful, except for two of our measurements of wildfire smoke at ~13.5 km^17^, due to the challenging conditions of very low CO mole fractions of 10–15 ppb^18^ and potential contamination during sampling^19^. Important questions regarding the isotope budget of CO concern the unknown oxygen isotope source signatures from CH_4_ oxidation both via the reaction of CH_3_+O_2_ and from O(^1^D) via R3b and R3c, and the importance of transport and oxidation by OH via R4.

Here we present the first in situ high altitude measurements of CO isotopes. We analyse 4 vertical profiles of the stable isotopic composition of CO based on 15 air samples collected between 12 and 25 km altitude using a lightweight stratospheric air (LISA) sampler on a weather balloon in Sodankylä, Finland^20^. This dataset allows us to explore the processes that cause the observed vertical distributions and infer the unknown source signatures of stratospheric CO originating from the photolysis of CO_2_. Furthermore, we use atmospheric transport and chemistry modelling to infer the isotope budget of CO, and confirm that oxygen with a very high ^18^O content is incorporated in a small fraction of the CO, originating from both O(^1^D) and CO_2_ photolysis.

## Results

### Vertical profiles of stratospheric δ^18^O-CO, δ^13^C-CO, CO and CH_4_

**Fig. 1:**
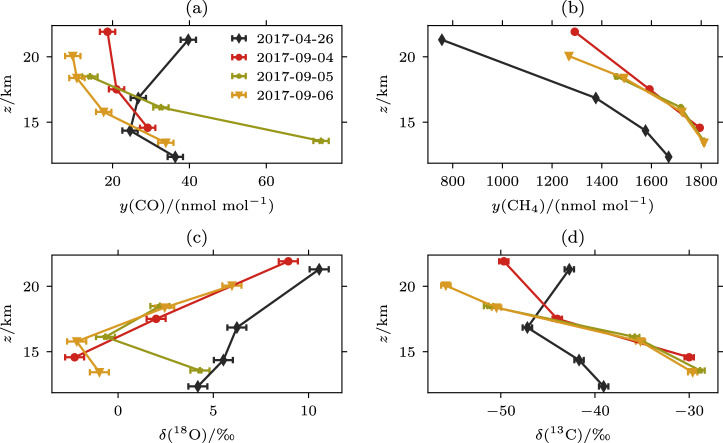
Vertical profiles of CO, CH_4_, δ^18^O-CO and δ^13^C-CO as function of altitude. Four vertical profiles of LISA sampler from balloon flights coloured by date: **a** CO mole fractions, **b** CH_4_ mole fractions, **c** δ^18^O-CO, (d) δ^13^C-CO. The uncertainties are reported as standard deviation (1*σ*), and are 2 ppb for CO, 3 ppb for CH_4_ and 0.5‰ for δ^18^O-CO and δ^13^C-CO, respectively. The uncertainties of CO and CH_4_ are estimated based on uncertainties associated with five terms: analysis, scale transfer, scale extrapolation, sampling dead volume and storage^37^. The uncertainties of δ^13^C-CO and δ^18^O-CO are estimated from the reproducibility of repeated sample measurements. The uncertainties are smaller than the symbol size for CH_4_ (**b**). Note that the profiles of CH_4_ (**b**) and δ^13^C-CO (**d**) for Sep 5 and Sep 6 are overlapping.

The observed vertical profiles of CO, CH_4_, δ^18^O-CO and δ^13^C-CO shown in Fig. 1 reveal generally decreasing trends with altitude for CO, CH_4_ and δ^13^C-CO and increasing trends for δ^18^O-CO. The vertical profiles of all species on 26 April 2017 are distinct from those obtained in September 2017, in particular through higher δ^18^O-CO and lower CH_4_ abundance. The low CH_4_ mole fraction observed on 26 April 2017 indicates the interception of air descending from high altitudes in the Arctic polar vortex, as reported previously^13^.

The mole fractions of CH_4_ and CO decrease with altitude due to their stratospheric sink reactions, R1–3 for CH_4_ and R4 for CO, respectively. The observed decrease of δ^13^C-CO with altitude can be explained by the inverse kinetic isotope effect in the CO sink, where ^13^CO reacts faster with OH than ^12^CO at low pressures^21^, and by the strongly ^13^C-depleted source from CH_4_ oxidation. Three features here, however, are unexpected. First, large variations among the four profiles in δ^13^C-CO, δ^18^O-CO and CO mole fractions are observed above 20 km. Secondly, increasing δ^18^O-CO values are observed at higher altitudes, although the oxidation of CH_4_ and the CO sink via OH are expected to yield low δ^18^O values^22^. Finally, there is a large enhancement of CO and δ^18^O-CO in the lowest part of the stratosphere (13.6 km sample) on 5 September. This feature results from wildfire smoke that was transported from British Columbia, Canada, into the stratosphere and was discussed in a previous publication^17^. The first two features will be elaborated on further in this paper. A comparison to earlier atmospheric observations is made in Supplementary Section 1.1.

### Source signatures of δ^18^O-CO and δ^13^C-CO originating from the photolysis of CO_2_

**Fig. 2:**
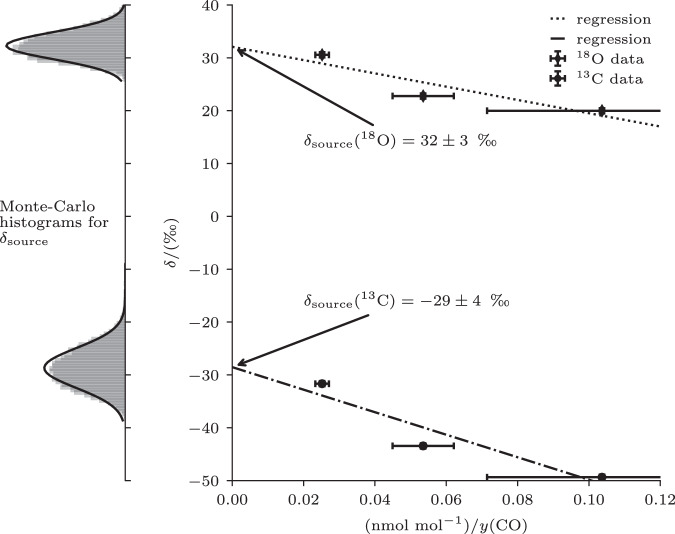
Determination of δ^18^O-CO and δ^13^C-CO source signatures for CO originating from the photolysis of CO_2_. The estimated isotopic composition of CO source mixture derived from measured δ^18^O-CO and δ^13^C-CO and corrected for OH sink isotope fractionation vs. the inverse CO mole fraction for the 3 uppermost samples of the dataset above 20 km (pressure < 60 hPa) from April 26, September 4 and 6, 2017. Ordinary least squares regression was performed on the data. The error bars represent the measurement uncertainty of 3 ppb for the CO mole fractions on the axis, with uncertainty propagation used to derive the 1/y(CO) uncertainty. The uncertainty in the CO isotope composition was 0.5 per mil. Both of these uncertainties, in addition to the uncertainty due to the OH correction, are used to estimate the uncertainty in the source signature shown on the PDFs on the left.

The enhancement of CO observed on 26 April 2017, and the changes in the vertical gradient in general at altitudes above 20 km (pressure < 60 hPa) in Fig. 1a are surprising features: the stratospheric production from methane oxidation cannot explain such large increases^23^. Meanwhile, deviations in the vertical gradient of δ^18^O-CO and δ^13^C-CO (Fig. 1c, d) is also visible. Using the atmospheric chemistry General Circulation Model EMAC (see “Methods” and Supplementary Section 7), we were able to reproduce this feature and attribute it to air transported from the mesosphere, where CO is produced from CO_2_ photolysis. A case of even stronger CO enhancements of up to 600 ppb, related to polar vortex dynamics, was studied before by Engel et al.^24^. Our measurements provide a unique opportunity to derive the isotopic source signatures of CO from mesospheric CO_2_ photolysis using the Keeling plot approach from the three uppermost samples, where this source is dominant. The derivation requires a correction of the integrated effect of isotope fractionation during CO oxidation by OH, which was obtained in a comprehensive simulation with the EMAC model. Ultimately, the obtained source signatures of δ^18^O-CO and δ^13^C-CO originating from the photolysis of CO_2_ are δ^18^O = 32 ± 2‰ and δ^13^C = −29 ± 3‰, respectively (see Fig. 2). Through a sensitivity test in the Monte Carlo simulations, we found that a 0.5 per mil uncertainty on the correction for OH fractionation yielded a 1.0 per mill additional uncertainty on the source signature estimate. These signatures indicate a different source than the most common stratospheric source from methane oxidation that would yield lower δ^13^C-CO and δ^18^O-CO.

Previous estimates of the isotopic source signature of CO from CO_2_ photolysis are based on either theoretical work^25^ or lab measurements at very narrow wavelength^26^, with neither case realistically representing stratospheric conditions. Our derived source signatures are in qualitative agreement with Schmidt et al.^25^, suggesting a substantial wavelength-dependent fractionation effect that depletes the CO product in both ^18^O and ^13^C compared to the CO_2_ substrate. More recently, a similar effect on the CO from photolysis of CO_2_ was found for the Martian atmosphere^27^.

Large amounts of CO derived from mesospheric CO_2_ photolysis occasionally enter the stratosphere near the polar vortex^24^. Our simulations suggest that this contribution to the stratospheric CO budget may be significant during summertime, which is consistent with the observed δ^13^C-CO and δ^18^O-CO at high altitudes. Examination of the CH_4_ mole fractions of those samples suggests that this air has not spent a significant amount of time in the mesosphere as this would yield very low CH_4_ mole fractions. The alternative hypothesis would be the mixture of air from the mesosphere and air that has recently transported to the stratosphere from the troposphere. This, could in principle explain the observed isotope composition, but requires physically implausible long transport timescales in the stratosphere. This leads us to conclude that the deviation of the trend of CO mole fractions with altitude at high altitudes, particularly visible on 26 April 2017, is caused by mixing of mesospheric air into the stratosphere, and that CO_2_ photolysis may be important for the stratospheric budget CO mole fractions and δ^18^O-CO.

### Origin of the high δ^18^O-CO: O(^1^D) and CO_2_ photolysis

Of particular interest is whether the obtained source signature of CO_2_ photolysis also explains the observed vertical gradient of δ^18^O-CO. An additional pathway of ^18^O -enriched CO production can be found by examining R3b and R3c. Here, the source of the oxygen is O(^1^D), which in the stratosphere is a product of ozone catalytic cycling, and is expected to have a very large ^18^O enrichment (~150‰) inherited from ozone^28^. Using our observations with the aid of the atmospheric model, we can derive the relative importance of the contributions from CO_2_ photolysis and O(^1^D) oxidation of CH_4_ to the final δ^18^O-CO values.

**Fig. 3:**
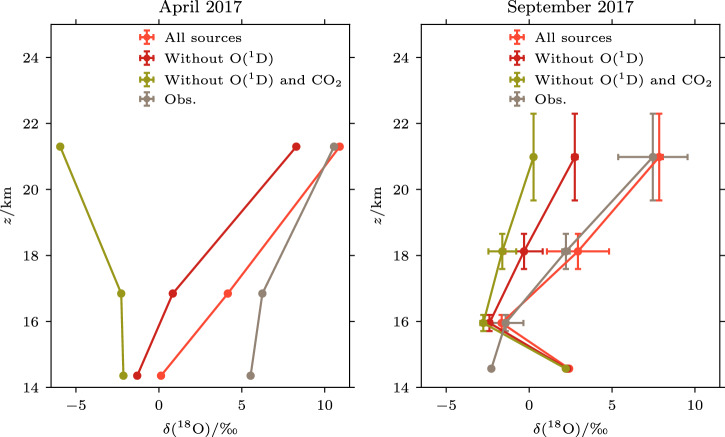
Comparison of observed and simulated profiles of δ^18^O-CO for the balloon flights in April 2017 (left) and in September 2017 (right). Simulation results based on the source signatures optimised with the profile observations are shown in orange, results omitting O(^1^D) in red and results omitting both O(^1^D) and CO_2_ photolysis in light yellow. In case a given CO source pathway is disregarded in the computation, it is assumed that its δ^18^O signature is the same as the one for the CH_3_+O_2_ pathway. The error bar of δ^18^O-CO for September 2017 indicates the standard deviation of the values of three profiles. No error bar is available for April 2017, as one profile does not allow for the calculation of the uncertainty.

Our analysis shows that the increasing δ^18^O-CO with altitude requires contributions from both the O(^1^D) reaction and CO_2_ photolysis (Fig. 3). Model simulations that include the O(^1^D) reaction and CO_2_ photolysis can reproduce well the observed δ^18^O-CO signal, except for the lowest, wildfire-influenced sample on 5 September^17^. When both O(^1^D) and CO_2_ photolysis are excluded in the model, the gradient of simulated δ^18^O with altitude becomes much weaker. The photolysis of CO_2_ affects mostly the samples at highest altitudes, as it is associated with the transport of air from the mesosphere. Also, the contribution of O(^1^D) to δ^18^O-CO is larger at high altitudes than at lower altitudes due to a larger contribution of CH_4_+O(^1^D) reaction to CO production (see Supplementary Fig. 2). Although this reaction is a minor contributor (up to ~5%) to the mole fraction of stratospheric CO, its influence on δ^18^O-CO is pronounced because the CO formed this way carries the very high ^18^O enrichment from O(^1^D) that originates from highly ^18^O-enriched ozone. Thus, the CO from the CH_4_+O(^1^D) reaction records unique information in its isotope composition, quantitively linking two important stratospheric processes, namely CH_4_ oxidation and the enrichment of ^18^O in O(^1^D) via ozone.

The observed isotope composition can only be reproduced in the model if we assume that δ^18^O signature of CO from CH_4_+O(^1^D) pathway is as large as 91‰ (see “Methods” and Table 1). It is expected that stratospheric O(^1^D) carries such a highly ^18^O-enriched signature because it is mainly produced from photolysis of ozone^29^. The δ^18^O-CO signature derived in this work is comparable to previously inferred δ^18^O of O(^1^D)^30^ and smaller than the estimated value of 150–160‰^28^, suggesting little room for a large fractionation during the photolysis reaction, as also argued by Zahn et al.^28^.

**Table 1:** Inferred isotopic signatures of major stratospheric CO sources

Sources of ^18^O or ^13^C in CO	*δ*	*u*(*δ*)^b^ 1*σ*
------Monte Carlo simulation------		
δ(^18^O) from CH_3_+O_2_	+15‰	6‰
δ(^18^O) from CH_4_+O(^1^D)^a^	+91‰	95‰
δ(^18^O) from CO_2_ photolysis	+34‰	3‰
------Keeling plot method------		
δ(^18^O) from CO_2_ photolysis	+32‰	2‰
δ(^13^C) from CO_2_ photolysis	−29‰	3‰

The uncertainties of the oxygen source signature, u(δ), are based on a Monte Carlo simulation (see Supplementary Section 6). The uncertainties of oxygen source signatures from Monte Carlo simulations and the uncertainties of both carbon and oxygen source signatures associated with CO_2_ photolysis are based on the Keeling plot approach.

^a^Including only the reactions that do not produce methyl radical (CH_3_).

^b^The uncertainties are reported as 1σ.

Our obtained signature for the reaction CH_3_+O_2_ agrees with the first estimate of Stevens et al.^31^ and also with estimates from an inverse modelling study^4^. However, it disagrees with earlier estimates of 0‰ derived from isotope budget closure using measurements from the Southern Hemisphere^32^. Our results can be reconciled with tropospheric estimates only if the effective sink isotope fractionation in CO oxidation by OH is underestimated by Brenninkmeijer et al.^32^.

Estimating the effect of oxidation of CO by the OH radical on the final CO isotope composition is intricate because of poor constraints on the key parameters defining it. For example, earlier studies assumed insignificance of the OH sink fractionation during an urban investigation^4^ or an equilibrium between CO sources and sinks^32^, which would allow for a simplified derivation. The inverse modelling studies using chemical transport and realistically varying OH fields disagree with these assumptions^12^, as do our results here. Therefore, we conclude that the sink fractionation effect of OH on CO isotopic composition may be significantly different from equilibrium fractionation and care has to be taken in quantifying its spatiotemporal distribution.

## Discussion

In the stratosphere, the sink of CH_4_ is a major source of water vapour, the key contributor to atmospheric radiative forcing, chemistry and dynamics^33^. However, the strength of stratospheric CH_4_ sink is yet subject to considerable uncertainty^34^, which complicates our understanding of the future change of stratospheric water vapour under various scenarios of atmospheric CH_4_ evolution.

Observations of CO and its isotopic composition can provide an additional way of evaluating the stratospheric CH_4_ sink and the abundance of OH via discernible influences of these on the δ^13^C and δ^18^O of the CO formed in the stratosphere. For example, with the reactions that capture the oxygen from O^1^D (R3b and R3c) accounting for only 25% of the overall reaction rate R3^35^, our model shows that a 4% increase in the CH_4_+O(^1^D) source will result in a 1‰ change in δ^18^O-CO; At the currently attainable CO stable isotope measurement uncertainty of 0.5‰, one can potentially distinguish changes on the order of 2% in the CH_4_+O(^1^D) sink rate. In contrast, a comparable change in the contributions of the other CH_4_ sink reactions, i.e. with reactants OH and Cl radicals, will not significantly alter the ^18^O content of CO. On the other hand, the reaction of CH_4_ with Cl is known to have a significant fractionation effect for ^13^C (ref. ^36^), thus small changes in Cl-driven sink may be well detectable in the δ^13^C of CO. CO_2_ photolysis has a significant effect on δ^18^O as well and can be identified through the co-variation of ^13^C-CO and CO mole fractions, with mutual increase indicating greater input share of CO_2_ photolysis source.

Our study demonstrates that subsidence of CO produced from mesospheric CO_2_ photolysis can best explain the observed enhancement of CO in the middle stratosphere, as shown by our model simulations and CO stable isotope observations. Simulations with the EMAC model demonstrate the ability of reproducing the combined chemical and dynamic nature of the observed CO isotope composition. The latter can be successfully employed for a range of atmospheric research problems dealing with trace gas chemistry and effects of CO_2_ on stratospheric and mesospheric dynamics.

Finally, laboratory studies that quantify the fractionation signatures of the oxygen isotopes from CO_2_ photolysis and CH_4_+O(^1^D) under a wide range of pressures and temperatures are encouraged. With the improved fractionation signatures and the isotopic composition measurements of CO and CO_2_ on routinely collected air samples using a lightweight stratospheric sampler^19^, CO stable isotopes will be a useful tracer for diagnosing important chemical reactions involving CH_4_, CO_2_ and H_2_O in the stratosphere.

## Methods

### Sample collection and analysis

Air samples were collected in Sodankylä (67.368°N, 26.633°E, 179 m.a.s.l.), Finland using a balloon-borne lightweight stratospheric air sampler as described in Hooghiem et al.^20^. A total of 15 air samples were collected on four individual days, i.e. on 26 April and 4–7 September 2017. The air samples were analysed for mole fractions of CO and CH_4_ using a cavity ring-down spectrometer in Sodankylä shortly after each flight. The CO and CH_4_ measurements are reported on the WMO X2014A and X2004A scales, with estimated uncertainties of 2 ppb and 3 ppb, respectively^37^. The air samples were then transferred and stored in glass flasks, and analysed 2–3 months after collection for δ^13^C-CO and δ^18^O-CO using continuous-flow isotope-ratio spectrometry at the Institute for Marine and Atmospheric research Utrecht (IMAU)^38^. The δ^13^C-CO and δ^18^O-CO measurements are reported relative to Vienna PeeDee Belemnite (VPDB) and Vienna Standard Mean Ocean Water (VSMOW), respectively, with estimated uncertainties of 0.5‰ for both. The bias on δ^13^C-CO measurements due to oxygen mass independent fractionation (MIF) caused by the sink reaction of CO and OH^39,40^ was estimated using the EMAC results to be less than 0.4‰, and is ignored.

### EMAC model simulations

Accurate estimates of the sink fractionation effect and transport of CO isotopologues require use of a comprehensive atmospheric chemistry transport model. Here we employ the EMAC (ECHAM/MESSy Atmospheric Chemistry) general circulation model^41^ to provide two pieces of information. First, the relative contribution of the CO surface sources and chemical sources/sinks was simulated to understand the CO budget and the global atmospheric distribution at the times of the observations performed by LISA. A kinetic tagging technique^42^ was used to trace the C and O exchanges between CO, CH_4_, CO_2_, O_3_, O(^1^D) including all intermediate carbon- and oxygen-bearing species to quantify pathways and contribution of elemental transfer to CO from its principal sources. The kinetic tagging allows distinguishing individual shares of C and O from CH_4_ oxidation, CO_2_ photolysis, the pathways involving O(^1^D), and inheritance of O from principal oxygen reservoirs (ozone, water vapour, molecular O_2_). Secondly, the magnitude of effective sink fractionation resulting from oxidation of CO by the OH radical and atmospheric transport was obtained.

EMAC simulations were performed at the T63L90MA resolution (middle atmosphere setup with model top at about 80 km, horizontal resolution of about 1.88°) with tropospheric dynamics weakly relaxed towards the analysed meteorology (ECMWF ERA-INTERIM reanalysis^43^). Relaxation allows close reproduction of realistic meteorological conditions in the troposphere, which is sufficient for accurately simulating stratospheric dynamics (e.g. polar vortex split and mesospheric air intrusions) as well^44^. Model and trace gas emission setup closely follow that evaluated in Lelieveld et al.^45^. The model was spun up for 10 years to ensure realistic circulation and tracer distribution in the stratosphere are reached by year 2017. The data for comparison and model-aided analysis was sampled along LISA flight tracks at the highest possible temporal resolution.

### Inferring isotopic source signatures

When two different air parcels mix, e.g. CO from a source added to background air, a simple linear relation exists between the inverse of the total mole fraction and the isotope ratio of the mixture expressed as δ value, known as the Keeling approach^46^. The intercept of the linear regression to the data in the δ - 1/CO space indicates the isotopic signature of the source. This approach is applied to the CO data for the samples collected at highest altitudes, i.e. enhanced CO from CO_2_ photolysis in the mesosphere, to obtain the CO source signature of CO_2_ photolysis. It is assumed, and confirmed by the EMAC simulations, that the effect of CO oxidation in the stratosphere was small over relevant timescales. Nevertheless, the fractionation effect would significantly bias the estimated source signature and therefore, a correction was applied before the regression analysis.

### Mass balance

An isotopic mass balance equation for CO with multiple sources and a sink in the stratosphere can be approximated as follows:

3 $${{\updelta }_{\mathrm{o}}=\,\mathop{\sum }\limits_{\mathrm{i}}{\mathrm{f}}_{\mathrm{i}}{\updelta }_{\mathrm{i}}+\varepsilon}$$

where *i* indicates the number of sources, *f*_*i*_ is the contribution of a specific source *i* with source signature *δ*_*i*_, and *ε* is the effective fractionation of the combined sink. With *δ*_*o*_ from the observations, and *f* and *ε* from the EMAC simulations, Eq. (3) can be used to infer the isotopic source signatures of δ_i_. A linear least squares algorithm was used to derive the source signatures while bounding the solutions based on observational constraints from earlier work. To assess the uncertainties associated with the simulated results by the EMAC and observations, a Monte-Carlo simulation was performed, yielding a close to normal estimate of the final uncertainty. Two important assumptions were made. First, the fractionation associated with sources and sink has no large temperature dependence. No data is available to verify or falsify this assumption. In our study, the stratospheric temperature for the collected air samples varies within 7 °C, and the fractionation deduced from the data is a mean value corresponding to a mean integrated temperature record of the sample in the stratosphere. Secondly, it is assumed that the dominant sources of O(^1^D) and O_2_ have vertically constant isotopic composition of oxygen. While this is an observed fact for O_2_^47^, it is close to a rough estimate for O(^1^D), which is expected to have some altitude dependence inherited from its dominant source O_3_. However, this is a second order effect on top of the high ^18^O enrichment, and some variation in δ^18^O may not be detectable with our current uncertainty.

## Supplementary information

Isotopic source signatures of stratospheric CO inferred from in situ vertical profiles

## Data Availability

The LISA data are available at 10.34894/YLXRBB.

## Code availability

The code is available at https://github.com/JJDHooghiem/JJDH_PhD_code.
