# Supplementary material for: Isotopic source signatures of stratospheric CO inferred from in situ vertical profiles
Source: NPJ Clim Atmos Sci. 2025 Mar 18;8(1):110. doi: 10.1038/s41612-025-00986-1 (PMC11919694; doi:10.1038/s41612-025-00986-1)
Supplement: Supplementary file 1 — Isotopic source signatures of stratospheric CO inferred from in situ vertical profiles [file 41612_2025_986_MOESM1_ESM.pdf]

Supplementary information

February 23, 2024

This document contains the supplementary material to “Isotopic source signatures of stratospheric CO inferred from in situ vertical profiles”.

1 Data comparison

A comparison with earlier observations of stable isotope composition of stratospheric CO is shown in Figure 1. In general, the CO obtained at similar potential temperature, in the stratosphere a proxy for both altitude and age, agrees with other data, in particular those obtained from the Caribic 1 campaign (S. Gromov and C. A. Brenninkmeijer, 2015). Our measurements in the lowermost stratosphere agree well with earlier data obtained from CARIBIC. The very depleted values, particularly in $\delta(^{18}\mathrm{CO})$, presented by Brenninkmeijer et al., 1996 are not observed in this work, which may be a feature of the Southern Hemisphere, where the tropospheric budget of CO is less influenced by the more enriched fossil fuel emissions, and depletion by OH may have been more important. This is supported by the effective fractionation computed in this work (see Figure S4) which shows that the depletion can be much larger than the equilibrium fractionation value commonly assumed.

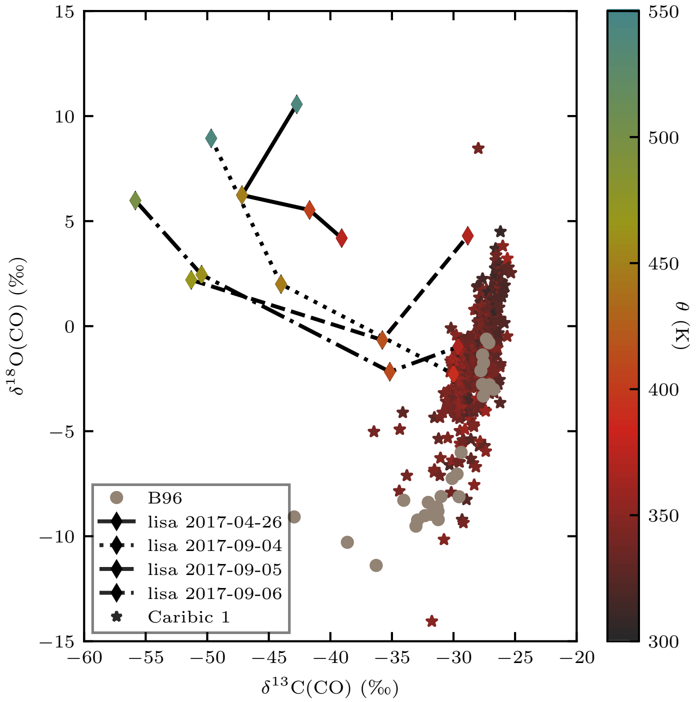

Figure S1 | Comparisons of LISA $\delta(^{18}CO)$ and $\delta(^{13}CO)$ observations with previous studies. The UTLS and stratospheric observations from various other campaigns. The shown CARIBIC 1 data is corrected for contamination (S. Gromov and C. A. Brenninkmeijer, 2015). The original data were contaminated but could be corrected for. Here only the corrected data are presented. The B96 measurements performed in the SH UTLS region (C. A. Brenninkmeijer et al., 1996) don't have potential temperature data and are therefore coloured grey.

2 Fractional contributions of CO budget

Figure 2 shows the simulated fractional contributions of the different sources to the CO at the sampling time and place of the LISA obtained observations. All stratospheric sources, CH_4, CO_2 become increasingly important, with increasing altitude. This is also the case for the relevance of $O(^1D)$ to the oxygen budget. The residual indicates still significant contribution of CO from the troposphere at low altitude. It is assumed that this fraction can be modelled with a single mean value.

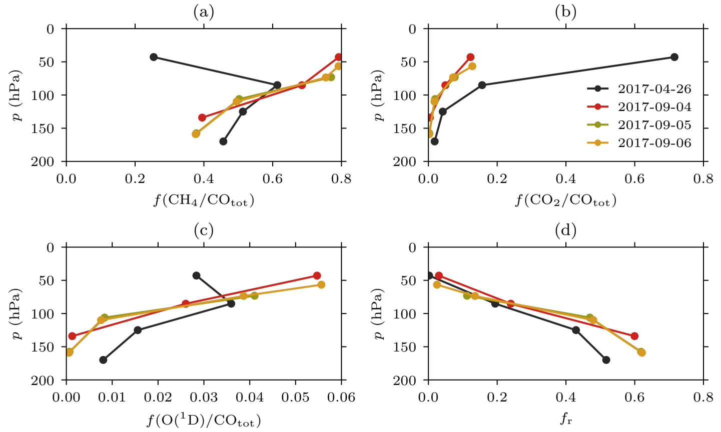

Figure S2 | Fraction of total CO resulting from the CH_4, CO_2, and $O(^1D)$ sources. Fractions of CH_4, CO_2, and $O(^1D)$ in CO, respectively panels a, b, and c. Panel d shows the residual which can be regarded as being exclusively produced in the troposphere. The model output is interpolated to the LISA sampler observations.

3 EMAC model setup

EMAC simulations were performed at the T63L90MA resolution (middle atmosphere setup with model top at about 80 km, horizontal resolution of about 1.88°) with tropospheric dynamics weakly relaxed towards the analysed meteorology (ECMWF ERA-INTERIM reanalysis, (Berrisford et al., 2011)). Relaxation allows close reproduction of realistic meteorological conditions in the troposphere, which is sufficient for accurately simulating stratospheric dynamics (e.g. polar vortex split and mesospheric air intrusions) as well (Jöckel et al., 2006). Model and trace gas emission setup closely follow that evaluated in (Lelieveld et al., 2016). The model was spun up for 10 years to ensure realistic circulation and tracer distribution in the stratosphere are reached by year 2017. The data for comparison and model-aided analysis was sampled along LISA flight tracks at the highest possible temporal resolution.

To understand the CO budget in the stratopshere, source strengths are obtained from EMAC. The source strengths are derived from simulation results from MECCA-TAG, a sub-model of the EMAC GCM (S. Gromov et al., 2010). S. Gromov et al., 2010 applied MECCA-TAG to stable isotopes in CO. However, the unknown fractionation factors, complicate direct modelling of the isotopic composition of CO in the atmosphere. Instead, MECCA-TAG is used in an approach similar to the approach presented by Frank et al., 2018. The amount of CH_4, CO_2, and $O(^1D)$ is traced into CO by introducing counterparts of the molecules involved in production of CO. This results in a doubled set of reactions, isolating the CO, and intermediates, produced from CO_2, CH_4, and $CH_4 + O(^1D)$. In this way, the source of one of the primary stratospheric precursors of CO can

be traced into CO. The fraction of a traced species can be obtained from the computed mole fractions x, or number densities n, as follows

$$f = \frac{n_{\text{tag}}(\text{CO})}{n(\text{CO})}, \tag{1}$$

where $n_{\text{tag}}(\text{CO})$ is the amount of CO the results from the isolated doubled chemical tagged system. Note that the doubled chemistry set does not interfere with regular chemistry.

The information from three tagged schemes was used. The first scheme traces CH_4-oxidative products. Secondly, CO resulting from CO_2 photolysis is tagged, to trace the amount of CO_2-derived CO. Finally, $O(^1D)$, which in the stratosphere is almost exclusively produced from ozone, is traced into CO separately, by tagging the reaction:

$$\text{CH}_4 + \text{O}(^1\text{D}) \longrightarrow \text{CH}_3\text{O} + \text{H}, \tag{1}$$

$$\text{CH}_4 + \text{O}(^1\text{D}) \longrightarrow \text{CH}_2\text{O} + \text{H}_2\cdot \tag{2}$$

It can be shown that other routes of ozone into CO are very small. Yet, the observations show an increasing enrichment in heavy oxygen with altitude, Figure 2, main manuscript. The main candidate for producing the observed enrichment is then Reactions 1 and 2. Hence, the separate tagged scheme is used for these reactions.

To distinguish between different tagged systems, the tagged quantities are given the subscript of the chemical species of interest. Thus, $f_{\text{CH}_4}(\text{CO})$ is the fraction of CO coming from CH_4 oxidation. Similarly, $f_{\text{O}(^1\text{D})}(\text{CO})$ is the CO that is produced in the reaction chain stemming from Reactions 1 and 2. A peculiarity arises due to recycling of tagged species, and overlap between tagged schemes. An obvious example $f_{\text{O}(^1\text{D})}(\text{CO}) \subset f_{\text{CH}_4}(\text{CO})$. Similarly, CO_2 produced in the CH_4 oxidation chain contains some $O(^1D)$ as well. This is then recycled into CO through CO_2 photolysis. The oxygen isotope composition of CO_2 is primarily controlled by the exchange reaction with $O(^1D)$ (Johnston et al., 2000; Lammerzahl et al., 2002; Thiemens et al., 1995; Wiegel et al., 2013; Yung et al., 1991). Thus, $O(^1D)$ containing CO is not of influence on the isotope composition of CO_2. Additionally, virtually all CH_3 is produced from CH_4 oxidation. Then, CH_3 reacts with oxygen. Thus, $f_{\text{O}_2}(\text{CO}) \approx f_{\text{CH}_4}(\text{CO})$, after $f_{\text{CH}_4}(\text{CO})$ is corrected for the contribution of $O(^1D)$. This gives the resulting corrections made:

$$f_{\text{O}(^1\text{D}),\text{cor}}(\text{CO}) = f_{\text{O}(^1\text{D})}(\text{CO}) - f_{\text{CO}_2}(\text{CO}) f_{\text{O}(^1\text{D})}(\text{CO}_2), \tag{2}$$

and

$$f_{\text{CH}_4,\text{cor}}(\text{CO}) = f_{\text{CH}_4}(\text{CO}) - f_{\text{O}(^1\text{D})}(\text{CO}) - f_{\text{CO}_2}(\text{CO}) f_{\text{CH}_4}(\text{CO}_2), \tag{3}$$

here $f_{\text{CH}_4,\text{cor}}(\text{CO})$ and $f_{\text{O}(^1\text{D}),\text{cor}}(\text{CO})$ are the corrected fractions of CH_4 and $O(^1D)$ in CO. Since the interest is in these fractions, the subscript cor will be omitted from here on. Note that the last term in Equation (2) is typically very

small. It's important to note that a small bias may exist in the correction factors related to CO_2; CO_2 derived CO is primarily produced in the mesosphere, where oxidation of CO_2 and $O(^1D)$ containing CO is continuing when air descents back into the stratosphere, since these corrections are typically very small, this bias is ignored. The model results are interpolated linearly using $x(CH_4)$ or p as the independent vertical coordinates.

The other sources, fossil fuel emissions, wildfire emissions, and NMHCs sources can be summarised in a residual as follows:

$$f_r = 1 - f_{O(^1D)}(CO) - f_{CO_2}(CO) - f_{CH_4}(CO). \tag{4}$$

The f_r term is exclusively produced in the troposphere, except for maybe a small amount of NMHCs. On the other hand, $f_{CH_4}(CO)$ is not necessarily exclusive to the stratosphere. In the troposphere, on average and approximately 0.33% of the CO is produced from CH_4 as well (S. Gromov et al., 2017).

In Figure 3 the fractions of the different sources are shown as vertical profiles.

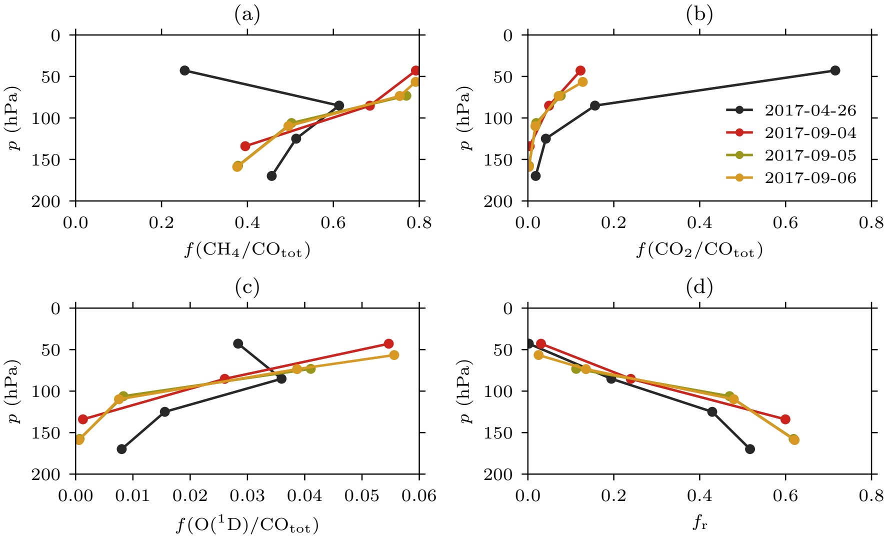

Figure S3 | The simulated relative contributions of the different in situ sources of CO. The profiles shown are sampled from the model at the observations. The vertical coordinate is the pressure at the observation.

4 Derivation of the mass balance

The mass balance of CO can be written in terms of the production P from different sources and its sink L_{OH}:

$$\sum_i P_i = L_{OH}. \tag{5}$$

Here i runs over all sources of CO, i.e. CH_4 oxidation, CO_2 photolysis, wildfire emissions, oxidation of non-methane hadrocarbons and fossil fuels. This equation applies to a steady state, i.e. production equals loss. Atmospheric CO is in general not in a steady state; It is assumed for now, and later it is qualitatively shown how to deal with non-steady state conditions for the isotope mass balance. Writing the mass balance for the stable isotopes is obtained from this by multiplying the source mass flux with the isotope amount fraction:

$$\sum_i P_i r_{\mathrm{sig},i} + = \frac{L_{(\mathrm{OH})}\alpha(\mathrm{OH})R(\mathrm{CO})}{1+\alpha(\mathrm{OH})R(\mathrm{CO})}. \tag{6}$$

Applying this to the oxygen enrichment, the isotope amount fraction is approximated by ignoring ^{17}O, and can be computed from the isotope ratio as follows:

$$^{18}r_i \approx \frac{^{18}R_{\mathrm{sig},i}}{1+^{18}R_{\mathrm{sig},i}}, \tag{7}$$

and on the right-hand side isotope ratio R by its source αR, where α is a correction for the isotope specific rate. The parametrisation of the fractionation in the CO sink, by OH, as used by (S. S. Gromov, 2013). The subscript "sig" is used to emphasise that the isotope ratio is the source signature and not the isotope enrichment of the source.

This implies that the fractionation in the processes that produce CO, is assumed to be independent of temperature and pressure. In addition, the source itself also must have a constant isotope enrichment. This may be appropriate to atmospheric oxygen, which has a constant atmospheric δ^{18}O value. In addition, O(^{1}D) likely has a very high δ^{18}O value. It is thus not unlikely that their source signatures are approximately constant. Currently, the kinetic isotope effects for the oxygen composition are assumed to be independent of temperature and pressure. Contrary to the in situ sources of carbon, the sources of oxygen in CO, have approximately constant enrichment.

To simplify the simulations with EMAC, and the computations, only three sources were considered explicitly. These are the important in-situ sources in the stratosphere: CO_2 photolysis, oxidation by CH_4 where oxygen is inherited from O_2, and oxidation by CH_4 where oxygen is inherited from O(^{1}D). A residual source term, denoted with a subscript r, which closes the mass balance implicitly accounts for the remaining sources. The possible range of the isotope enrichment is discussed later on. The composition of the CO in terms of its sources depends on the relative strength of the fluxes, for example the fraction f_{CH_4} of CH_4 in CO:

$$f_{\mathrm{CH}_4} = \frac{P_{\mathrm{CH}_4}}{P_{\mathrm{CO}_2}+P_{\mathrm{CH}_4}+P_{\mathrm{O}(^1\mathrm{D})}+P_{\mathrm{r}}} = \frac{P_{\mathrm{CH}_4}}{L_{\mathrm{OH}}}. \tag{8}$$

Substitution of this gives equation Equation (6) in terms of the amount of CO from relative fractions:

$$\frac{f_{\mathrm{CO}_2}R_{\mathrm{sig,CO}_2}}{1+R_{\mathrm{sig,CO}_2}} + \frac{f_{\mathrm{CH}_4}R_{\mathrm{sig,CH}_4}}{1+R_{\mathrm{sig,CH}_4}} + \frac{f_{\mathrm{O}(^1\mathrm{D})}R_{\mathrm{sig,O}(^1\mathrm{D})}}{1+R_{\mathrm{sig,O}(^1\mathrm{D})}} + \frac{f_{\mathrm{r}}R_{\mathrm{r}}}{1+R_{\mathrm{r}}} = \frac{\alpha(\mathrm{OH})R(\mathrm{CO})}{1+\alpha(\mathrm{OH})R(\mathrm{CO})}. \tag{9}$$

The isotope ratio $R(\text{CO})$ can be derived from the individual observations made by the LISA sampler, making use of the definition of the δ-value and the value of the isotope ratio of the reference material $^{18}R_{\text{VSMOW}} = 0.0020052$ (Coplen et al., 2002).

It is good to point out that in Equation (9) the term-specific denominator $1 + R$ is close to unity and does not differ too much between the terms, owing to the fact that isotope abundance variation amongst materials is small. In literature, it is often assumed that these are all equal, so that the disappear from the denominator, which then yields an equation of the form (check out the notation used by e.g. Bergamaschi et al., 2000; Manning et al., 1997):

$$\sum_i f_i \delta_i + \varepsilon = \delta_{\text{o}}, \tag{10}$$

where ε is the enrichment in the reaction with OH.

If no sink fractionation occurred, $\alpha(\text{OH}) = 1$, then the assumption that a steady-state exist, would not have been necessary. The observed enrichment would always be determined by the relative source strengths, e.g. end-member mixing. Since there is significant fractionation from the sink reaction, however, any attempt to obtain source signatures from stable isotope observations in the atmosphere, is nearly impossible due to the a priori unknown size of the sink fractionation. An alternative method to obtain the size of the sink fractionation was used in a 3D simulation by Manning et al., 1997 and Bergamaschi et al., 2000. A CO counterpart is emitted or produced at the same rate as CO, thus exactly the same as normal CO. This counterpart is subjected to the same transport and chemistry as CO, with one exception: the reaction of CO with OH occurs at the rate of the minor isotopologue of interest. Since, each isotopologue is subjected to a different fractionation, a counterpart for each isotopologue is required. Initially, the counterpart has an abundance equal to CO, $n_{\text{c}}(\text{CO}) = n(\text{CO})$, c denoting the counterpart. This is equivalent to an emission with an isotope ratio of $R_{\text{source}} = 1$. At first this, this may appear somewhat arbitrary. However, this justified since the only factor changing the isotope ratio is the removal by OH. Then, the enrichment, $\varepsilon(x, t)$ at location x and t is:

$$\varepsilon(x, t) = \frac{R(x, t)}{R_{\text{source}}} - 1 = \frac{n_{\text{c}}(\text{CO})}{n(\text{CO})} - 1. \tag{11}$$

This method singles out the total effect of the sink reaction, and hence is called the effective sink fractionation. When more CO is produced than oxidised, ε decreases, and vice versa. As pointed out by Bergamaschi et al., 2000, this approach is independent of the source signature of the emission, which is illustrated above by the independence of the choice for R_{source}.

Now, it has to be stressed that the model does not provide an estimate of the true value of $R(x, t)$. However, the measurements do provide the true value. The original mix of sources, i.e. not fractionated due to removal by OH, can be obtained as followed. With $R(x, t)$ provided by the measurement, and $\varepsilon(x, t)$ obtained from the simulations, an accurate value of R_{sources} can be obtained

from the definition of ε. Here the subscript was changed deliberately from source to sources, to emphasise the fact that this variable in the presented method is a mix of the different sources of CO. In terms of the δ-values and the enrichment, this becomes:

$$R_{\text{sources}} = R_{\text{std}} \left(\frac{\delta_{\text{o}} + 1}{\varepsilon + 1} \right), \tag{12}$$

note that the absolute values of δ and ε are used, rather than the values expressed in per mille. This approach effectively removes the effect that the sink had on the observed isotope enrichment. As mentioned, in this work a single model variable is introduced to trace the effective fractionation for the isotopologues ^{13}CO, $C^{17}O$, and $C^{18}O$. This is different from Bergamaschi et al., 2000 where a single variable for each individual source was introduced, and only ^{13}CO and $C^{18}O$ were traced. Finally, although steady-state was assumed to derive Equation (10), by making use of the effective sink fractionation, this is no longer a requirement.

Figure 4 shows the simulated effective fractionation profile. As a reference, the equilibrium fractionation profile is presented as well. Comparing these two it is clear that depletion of CO found in the air samples is well beyond equilibrium. Furthermore, seasonal and vertical variation is clearly visible. This complicates the use of simple assumption to derive source signatures.

5 Minimum least-squares optimization of the oxygen source signatures of CO sources

Equation (9) can be applied individually to each of the 11 observations made by the LISA sampler, resulting in a set of 11 equations. $\alpha(\text{OH})R(\text{CO})$ in Equation (9) is replaced with the isotope ratio of the observation, corrected for the effective sink fractionation as explained above. Such a linear system can be put into a matrix equation of the following form

$$Ab = x. \tag{13}$$

In Equation (13) b is a vector of length N, with N the number of source signatures, and the elements of b are numbers that can be mapped to the source signatures. x is a vector of length M, M is the number of observations, provided by the LISA sampler (δ-values). A is a matrix of $\dim(M, N)$ containing the f's provided by the MECCA-TAG EMAC simulation results to solve for b using a bounded-value least-squares method (Stark and Parker, 1995). Note that a summation of a row in of A should equal 1 to conserve mass:

$$\sum_j A_{ij} = 1, \tag{14}$$

The residual term is in principle different for each observation used in the inversion. The term will depend on the relative importance of the remaining sources and can thus be constrained using data presented by (Vimont et al., 2019).

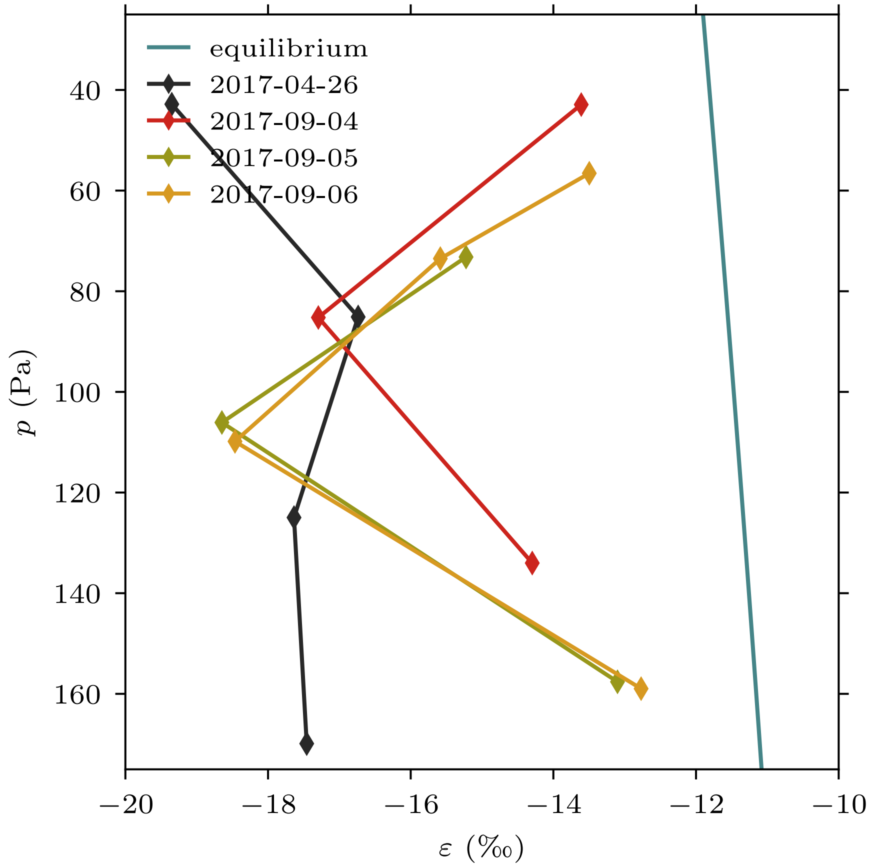

Figure S4 | Simulated profiles of the effective fractionation. The profile for equilibrium is included as a reference.

The highest enrichment was found for fossil fuel combustion, with $\delta = 23.5‰$. A lower enrichment was found for NMHCs, possibly as low as 0‰. When NMHCs are oxidised by ozone, the $\delta^{18}O$ values can be high, 100‰ (Röckmann, C. A. M. Brenninkmeijer, Neeb, et al., 1998), but its contribution was believed to be small (Huff and Thiemens, 1998; Röckmann, C. A. M. Brenninkmeijer, Saueressig, et al., 1998). Adding to that, the mean atmospheric budget suggests that the relative importance of these source to the residual term is not more than 50% (S. Gromov et al., 2017) on average. It is thus reasonable to assume that the residual term lies in the range 0–24‰. The limits can be constraint further, since it is unlikely that air in the free troposphere and stratosphere consists of only a single source. However, it turns out that the residual term will not be the limiting constraint to the inversion for the most likely scenarios.

In addition to the constraints on the residual term mentioned in the previous paragraph, a constraint can also be put on the source signature of CH_4 that subsequently results in the reaction $CH_3 + O_2$. Several attempts have been made to obtain a source signature for this reaction (C. A. M. Brenninkmeijer and Röckmann, 1997; Stevens and Wagner, 1989). Analysis of the budgets suggests that it would be as low a 0‰. Inverse modelling by Bergamaschi et al., 2000 estimated consistently higher values, around 9‰ and the earlier work by Stevens and Wagner, 1989, yielded a value close to 15‰. Therefore, in addition to inversions with an unconstrained CH_4 signature, CH_4 is constrained to be within 0–15‰.

The mix of the residual term, may however be different for different samples obtained by the LISA sampler. A couple of scenarios is investigated. First, a computation was performed with one simple parameter, f_r, to ensure $\sum_i f_i = 1$. In a second scenario, a single parameter for each season, i.e., April and September. Motivation for this model can be found in the tape recorder that is found in the stratospheric tropical pipe from the Microwave Limb Sounder, and which is related to the different biomass burning seasons in the tropics (Schoeberl et al., 2006). The final computation uses a parameter that was introduced for each of the three different isentropic surfaces that the observations were made on. The motivation of such a model is provided by the fact that air is transported from the tropics to the poles on isentropic surfaces mainly.

More parameters generally lead to unrealistic δ-values ($900 >|(\delta)|$), and when constrained no difference is observed from the models already introduced. Scenarios that were tried, are a combination of vertical and seasonal parameters, thus allowing for six extra parameters to be optimised. Finally, a sample-specific residual term was introduced for every observation made, which is 11, leading to the only under constrained optimisation that was investigated. The latter obviously results in minimal residuals. Yet, the solutions for $O(^1D)$, CO_2, and CH_4 derived CO are nearly identical, and the observed variability and vertical gradient are entirely attributed to the residual mass fractions. Moreover, the values obtained cannot be explained with the known source signatures.

In summary, the previous paragraphs describe the three scenarios considered in the inverse computation. These scenarios are summarised in Table 1. In total three scenarios are considered for the residual term.

Table S1 | The six scenarios used in the inversion. The residual column shows the amount of residual terms used and the rule column how a single residual term for an observations is distributed between the different terms introduced.

Scenario	residual	rule	$CH_3 + O_2$ constraint
S1	1		0–15‰
S2	2	seasonal	0–15‰
S3	3	altitude	0–15‰

6 Uncertainty analysis

Here the uncertainty analyses for obtained signatures is presented. First, the uncertainty associated with in the analytical uncertainty, $\sigma_\mathrm{a} = 0.5‰$ for δ^{18}O observations is assessed by means of a Monte-Carlo simulation. The enrichment is randomly drawn from a normal distribution whose standard deviation is 0.5‰ i.e. the assigned measurement uncertainty. This is determined for each observation individually, the gradient is thus allowed to change. The inversion was then repeated 10^6 times, and the resulting standard deviation is computed for the optimised source signatures. The simulation was performed for the scenario with one residual term, S1 Table 1. Two computations were made, with and without constraints on all of the terms. Constraints may result in an underestimate of the standard deviation and thus in a bias.

Secondly, it was shown that obtained CO stable isotope data may suffer from a bias due to storage (Hooghiem et al., 2020). Since the nature of underlying principles are unknown, it is difficult to find a reasonable constraint on the bias. To elucidate the effect of the bias on the obtained source signatures the following was considered. It is assumed that the bias on is from a Gaussian distribution. A constant systematic bias, i.e. the mean bias, in the measurements results in an equally large systematic bias in the derived source signatures. This can be shown mathematically: $\sum_j A_{ij}x_j = b_i$; Suppose $\sum_j A_{ij}x'_j = b'_i$ with $b'_i = b_i + c$, c is a constant. Also, $x'_j = x_j + d$, with d a constant. Then, $\sum_j A_{ij}x'_j = \sum_j A_{ij}x_j + \sum_j A_{ij}d = b + \sum_j A_{ij}d$. From this it follows that, $d\sum_j A_{ij} = c$ and because of Equation (13), $c = d$. Further, the variability in the bias can be added to the analytical uncertainty described above. It was verified that the obtained uncertainty for the source signatures scale with the input uncertainty, i.e. if the input uncertainty is $2\sigma_\mathrm{a}$, then the final uncertainty is also twice as large compared to computations with no bias.

Then, A modified one-parameter-at-a-time sensitivity test was performed to assess the response of the model to 10% increase or decrease of a column in A. Mass conservation prevents a true one-parameter-at-a-time analysis, as reduction or increase of a fraction must be compensated for. This is achieved by increasing or decreasing the residual term. The scenario for the residual term used, is the simple scenario for simplicity. Again, no constraints were used in the sensitivity test.

Finally, in order to obtain an estimate of the presented source signatures, the sensitivity test and the analytical uncertainty is combined in a second Monte-Carlo simulation. Where the analytical uncertainty was included as described above, the uncertainty in the fractions was included as follows. The fractions, f_X, are randomly varied from a Gaussian distribution with a standard deviation of 10% of their value, i.e. $\sigma = 0.1 f_\mathrm{X}$. Here X is one of the species CH_4, O(^{1}D), or CO_2. All fractions are allowed to change a little bit in a single Monte-Carlo experiment. The residual term is calculated as in Equation (4), and all fractions should should always be within $0 \leq f \leq 1$, thus mass is conserved. Scenario S1 is used in the uncertainty analysis.

7 CO_2 photolysis from the mesosphere

In Figure 5 the mole fractions of CO are shown from the EMAC model simulations. In Figure 6, the relative contribution of CO_2 photolysis is highlighted, suggesting the dominant source of the additional CO is this reaction. This photolysis reaction is the most likely explanation for the observed isotope observations and mole fractions. This would imply that CO2 photolysis is an important component in the stratospheric budget of CO, and may not be ignored in atmospheric composition studies of the stratosphere.

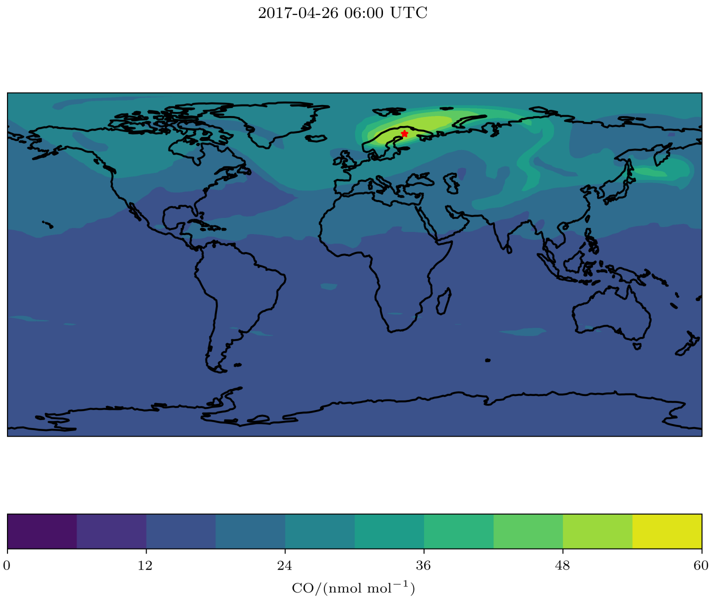

Figure S5 | Global map of CO at model level 15, approximately corresponding to the altitude of the observation indicated by the red start. The maximum mole fractions can be found over northern Europe.

References

Bergamaschi, P., R. Hein, C. A. M. Brenninkmeijer, and P. J. Crutzen (2000). "Inverse modeling of the global CO cycle: 2. Inversion of $^{13}C/^{12}C$ and $^{18}O/^{16}O$ isotope ratios". In: *Journal of Geophysical Research: Atmospheres* 105.D2, pp. 1929–1945. DOI: 10.1029/1999JD900819.

Berrisford, P., D. Dee, P. Poli, R. Brugge, M. Fielding, M. Fuentes, P. Kållberg, S. Kobayashi, S. Uppala, and A. Simmons (2011). *The ERA-Interim archive Version 2.0.* 1. ECMWF, p. 23.

Brenninkmeijer, C. A. M. and T. Röckmann (1997). "Principal factors determining the $^{18}O/^{16}O$ ratio of atmospheric CO as derived from observations in the southern hemispheric troposphere and lowermost stratosphere". In: *Journal of Geophysical Research: Atmospheres* 102.D21, pp. 25477–25485. DOI: 10.1029/97JD02291.

2017-04-26 06:00 UTC

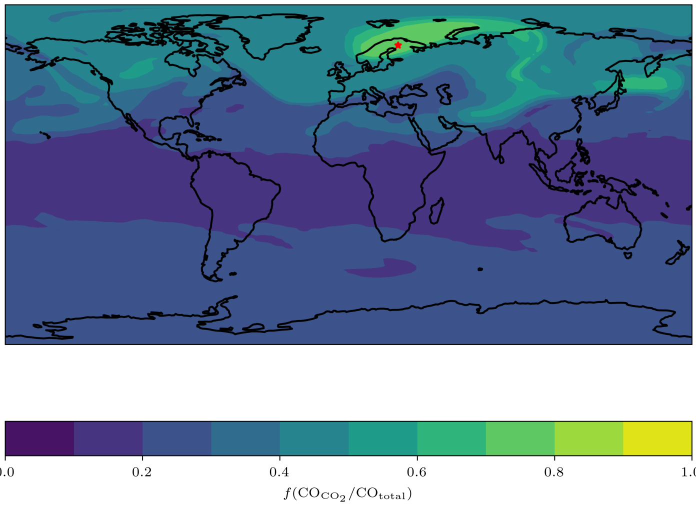

Figure S6 | Same as Figure 5, but now showing the fraction of CO which results from CO_2 photolysis. The plume correlates well with the observed CO. The map also indicates that CO_2 photolysis can be a significant component of the stratospheric budget of CO.

Brenninkmeijer, C. A., R. Müller, P. J. Crutzen, D. C. Lowe, M. R. Manning, R. J. Sparks, and P. F. J. van Velthoven (1996). "A large ^{13}CO deficit in the lower Antarctic stratosphere due to "Ozone Hole" Chemistry: Part I, Observations". In: *Geophysical Research Letters* 23.16, pp. 2125–2128. DOI: 10.1029/96GL01471.

Coplen, T. B., J. a. Hopple, J. K. Böhlke, H. S. Peiser, S. E. Rieder, H. R. Krouse, K. J. R. Rosman, T. Ding, R. D. J. Vocke, K. M. Révész, a. Lamberty, P. Taylor, and P. D. Bièvre (2002). "Compilation of minimum and maximum isotope ratios of selected elements in naturally occurring terrestrial materials and reagents". In: *Usgs*, p. 110.

Frank, F., P. Jöckel, S. Gromov, and M. Dameris (2018). "Investigating the yield of H_2O and H_2 from methane oxidation in the stratosphere". In: *Atmospheric Chemistry and Physics* 18.13, pp. 9955–9973. DOI: 10.5194/acp-18-9955-2018.

Gromov, S. and C. A. Brenninkmeijer (2015). "An estimation of the $^{18}O/^{16}O$ ratio of UT/LMS ozone based on artefact CO in air sampled during CARIBIC flights". In: *Atmospheric Chemistry and Physics* 15.4, pp. 1901–1912. DOI: 10.5194/acp-15-1901-2015.

Gromov, S., C. A. Brenninkmeijer, and P. Jöckel (2017). "Uncertainties of fluxes and ^{13}C / ^{12}C ratios of atmospheric reactive-gas emissions". In: *Atmospheric Chemistry and Physics* 17.13, pp. 8525–8552. DOI: 10.5194/acp-17-8525-2017.

Gromov, S., P. Jöckel, R. Sander, and A. W. Brewer (2010). "Geoscientific Model Development A kinetic chemistry tagging technique and its application to modelling the stable isotopic composition of atmospheric trace gases". In: *Geosci. Model Dev* 3, pp. 337–364. DOI: 10.5194/gmd-3-337-2010.

Gromov, S. S. (2013). "Stable isotope composition of atmospheric carbon monoxide: A modelling study". PhD thesis. Johannes Gutenberg Universität Mainz. DOI: 10.13140/RG.2.2.30769.17760.

Hooghiem, J. J. D., M. E. Popa, T. Röckmann, J.-U. Grooß, I. Tritscher, R. Müller, R. Kivi, and H. Chen (2020). "Wildfire smoke in the lower stratosphere identified by in situ CO observations". In: *Atmospheric Chemistry and Physics* 20.22, pp. 13985–14003. DOI: 10.5194/acp-20-13985-2020.

Huff, A. K. and M. H. Thiemens (1998). "$^{17}O/^{16}O$ and $^{18}O/^{16}O$ isotope measurements of atmospheric carbon monoxide and its sources". In: *Geophysical Research Letters* 25.18, pp. 3509–3512. DOI: 10.1029/98GL02603.

Jöckel, P., H. Tost, A. Pozzer, C. Brühl, J. Buchholz, L. Ganzeveld, P. Hoor, A. Kerkweg, M. G. Lawrence, R. Sander, B. Steil, G. Stiller, M. Tanarhte, D. Taraborrelli, J. van Aardenne, and J. Lelieveld (2006). "The atmospheric chemistry general circulation model ECHAM5/MESSy1: consistent simulation of ozone from the surface to the mesosphere". In: *Atmospheric Chemistry and Physics* 6.12, pp. 5067–5104. DOI: 10.5194/acp-6-5067-2006.

Johnston, J. C., T. Röckmann, and C. A. M. Brenninkmeijer (2000). "CO_2 +O(^{1}D) isotopic exchange: Laboratory and modeling studies". In: *Journal of Geophysical Research: Atmospheres* 105.D12, pp. 15213–15229. DOI: 10.1029/2000JD900070.

Lammerzahl, P., T. Röckmann, C. A. Brenninkmeijer, D. Krankowsky, and K. Mauersberger (2002). "Oxygen isotope composition of stratospheric carbon dioxide". In: *Geophysical Research Letters* 29.12, pp. 2–5. DOI: 1582\rArtn1582.

Lelieveld, J., S. Gromov, A. Pozzer, and D. Taraborrelli (2016). "Global tropospheric hydroxyl distribution, budget and reactivity". In: *Atmospheric Chemistry and Physics* 16.19, pp. 12477–12493. DOI: 10.5194/acp-16-12477-2016.

Manning, M. R., C. A. M. Brenninkmeijer, and W. Allan (1997). "Atmospheric carbon monoxide budget of the southern hemisphere: Implications of $^{13}C/^{12}C$ measurements". In: *Journal of Geophysical Research: Atmospheres* 102.D9, pp. 10673–10682. DOI: 10.1029/96JD02743.

Röckmann, T., C. A. M. Brenninkmeijer, P. Neeb, and P. J. Crutzen (1998). "Ozonolysis of nonmethane hydrocarbons as a source of the observed mass independent oxygen isotope enrichment in tropospheric CO". In: *Journal of Geophysical Research: Atmospheres* 103.D1, pp. 1463–1470. DOI: 10.1029/97JD02929.

Röckmann, T., C. A. M. Brenninkmeijer, G. Saueressig, P. Bergamaschi, J. N. Crowley, H. Fischer, and P. J. Crutzen (1998). "Mass-Independent Oxygen Isotope Fractionation in Atmospheric CO as a Result of the Reaction CO + OH". In: *Science* 281.5376, pp. 544–546. DOI: 10.1126/science.281.5376.544.

Schoeberl, M. R., B. N. Duncan, A. R. Douglass, J. Waters, N. Livesey, W. Read, and M. Filipiak (2006). "The carbon monoxide tape recorder". In: *Geophysical Research Letters* 33.12. DOI: https://doi.org/10.1029/2006GL026178.

Stark, P. B. and R. L. Parker (1995). "Bounded-variable least-squares: an algorithm and applications". In: *Computational Statistics* 10.

Stevens, C. M. and A. F. Wagner (1989). "The Role of Isotope Fractionation Effects in Atmospheric Chemistry". In: *Zeitschrift für Naturforschung A* 44.5, pp. 376–384. DOI: 10.1515/zna-1989-0505.

Thiemens, M. H., T. L. Jackson, and C. A. Brenninkmeijer (1995). "Observation of a mass independent oxygen isotopic composition in terrestrial stratospheric CO_2, the link to ozone chemistry, and the possible occurrence in the Martian atmosphere". In: *Geophysical Research Letters* 22.3, pp. 255–257. DOI: 10.1029/94GL02996.

Vimont, I. J., J. C. Turnbull, V. V. Petrenko, P. F. Place, C. Sweeney, N. Miles, S. Richardson, B. H. Vaughn, and J. W. C. White (2019). "An improved estimate for the $\delta^{13}C$ and $\delta^{18}O$ signatures of carbon monoxide produced from atmospheric oxidation of volatile organic compounds". In: *Atmospheric Chemistry and Physics* 19.13, pp. 8547–8562. DOI: 10.5194/acp-19-8547-2019.

Wiegel, A. A., A. S. Cole, K. J. Hoag, E. L. Atlas, S. M. Schauffler, and K. A. Boering (2013). "Unexpected variations in the triple oxygen isotope composition of stratospheric carbon dioxide". In: *Proceedings of the National Academy of Sciences of the United States of America* 110.44, pp. 17680–17685. DOI: 10.1073/pnas.1213082110.

Yung, Y. L., W. B. DeMore, and J. P. Pinto (1991). "Isotopic exchange between carbon dioxide and ozone via O(1D) in the stratosphere". In: *Geophysical Research Letters* 18.1, pp. 13–16. DOI: https://doi.org/10.1029/90GL02478.
